# Development of Molecularly Imprinted Polymers for Fenthion Detection in Food and Soil Samples

**DOI:** 10.3390/nano12132129

**Published:** 2022-06-21

**Authors:** Saqib Farooq, Bochang Chen, Fukun Gao, Ihsan Muhammad, Shakeel Ahmad, Haiyan Wu

**Affiliations:** Guangxi Key Laboratory of Agric-Environment and Agric-Products Safety, Agricultural College of Guangxi University, Nanning 530004, China; saqibhort@gmail.com (S.F.); 15677802394@163.com (B.C.); 18854881887@163.com (F.G.); ihsanagrarian@yahoo.com (I.M.); shakeel1287@hotmail.com (S.A.)

**Keywords:** fenthion, molecularly imprinted polymers, room-temperature ionic liquid, adsorption, reusability, real samples

## Abstract

Modern agricultural production is greatly dependent on pesticide usage, which results in severe environmental pollution, health risks and degraded food quality and safety. Molecularly imprinted polymers are one of the most prominent approaches for the detection of pesticide residues in food and environmental samples. In this research, we prepared molecularly imprinted polymers for fenthion detection by using beta-cyclodextrin as a functional monomer and a room-temperature ionic liquid as a cosolvent. The characterization of the developed polymers was carried out. The polymers synthesized by using the room-temperature ionic liquid as the cosolvent had a good adsorption efficiency of 26.85 mg g^−1^, with a short adsorption equilibrium time of 20 min, and the results fitted well with the Langmuir isotherm model and pseudo-second-order kinetic model. The polymer showed cross-selectivity for methyl-parathion, but it had a higher selectivity as compared to acetamiprid and abamectin. A recovery of 87.44–101.25% with a limit of detection of 0.04 mg L^−1^ and a relative standard deviation of below 3% was achieved from soil, lettuce and grape samples, within the linear range of 0.02–3.0 mg L^−1^, using high-performance liquid chromatography with an ultraviolet detector. Based on the results, we propose a new, convenient and practical analytical method for fenthion detection in real samples using improved imprinted polymers with room-temperature ionic liquid.

## 1. Introduction

**Figure 1:**
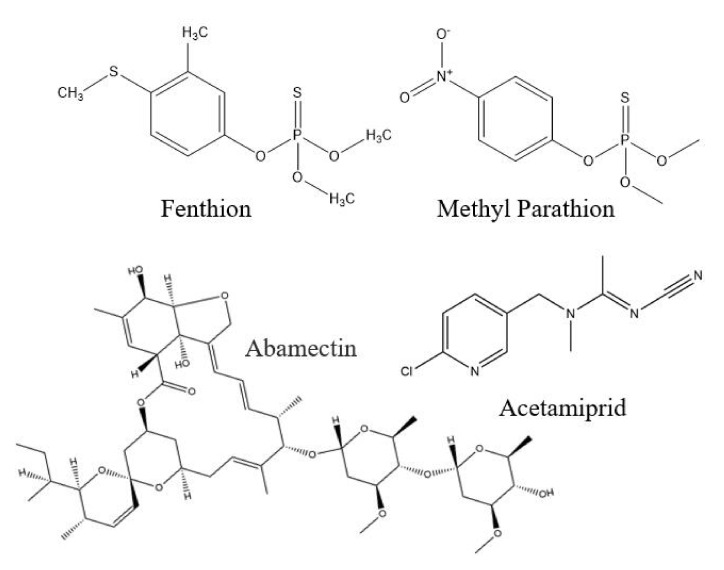
Chemical structure of fenthion, methyl parathion, abamectin and acetamiprid.

Global agricultural production is greatly reliant on pesticides and fertilizers to secure sufficient food production for society; however, as a consequence, environmental pollution and food quality and safety have been revealed as prominent issues. These emerging problems need to be addressed regularly with appropriate strategies. Fenthion (FNT) (Figure 1) belongs to the family of organophosphorus pesticides, and it is used as an insecticide in different fruit and vegetable crops [1]. The phosphoryl group of FNT forms phosphorylated cholinesterase by interacting covalently with the agile enzymatic part, thus causing the accumulation of acetylcholine in the body, leading to neurological disorders and intermediate syndromes [2]. FNT is a slow-degrading compound that has prominent toxic effects on birds [3]. Pesticide residue intake is greater with fresh food such as fruits and vegetables, which are used directly, resulting in severe negative effects [4]. Several methods have been introduced for the detection of FNT, such as gas chromatography with flame photometric detector (GC-FPD) [1], high-performance liquid chromatography with ultraviolet (HPLC–UV) [3], GC–tandem mass spectrometry (GC–MS/MS) [5] and liquid chromatography–tandem mass spectrometry (LC–MS/MS) [6]. However, traditional FNT detection with these methods has several limitations, such as arduous cleanup steps due to the complex matrix, large time requirements or lower detection ability.

In response to regular pesticide application in agriculture, new, rapid analytical methods are required for pesticide detection with easy application, low costs and reliability [7]. Molecular imprinting technology is a well-known approach for pesticide residue detection, involving the synthesis of molecularly imprinted polymers (MIPs) [8]. The synthesized imprinted polymers have specific template-oriented recognition sites that are developed through polymerization. Generally, MIPs are cost-effective, rapid, selective to a specific analyte, have high thermo-chemo stability and have wide application to various fields [9]. The performance and effectiveness of MIPs are hindered because they possess some limitations, which include their limited application and applicability and complex synthesis [10].

Due to wide availability, low cost, wide application and environmentally friendly nature, cyclodextrins are often selected as functional monomers for MIP synthesis. Beta-cyclodextrin (β-CD), with its hydrophobic interior and opposing exterior, forms an easily reversible guest–host interaction in polymerization, which results in improved MIP synthesis [11]. Dimethyl sulfoxide (DMSO) is usually used as a solvent for β-CD-based MIPs, but it results in poor MIP production because DMSO provides a poor medium during polymerization. Such a limitation can be addressed with room-temperature ionic liquids (RTILs), as they are known as greener and more effective solvents [12]. In addition to their various properties, such as thermostability, eco-friendly nature and wide applicability, they can boost MIPs’ efficiency due to their tunable nature and ionic interaction [13,14]. Using RTILs as cosolvents, molecularly imprinted polymers were previously prepared for the selective extraction of abamectin [15]. Moreover, 1-butyl-3-methylimidazolium tetrafluoroborate ([BMIM]BF_4_) is an ionic liquid widely used as a solvent in the polymerization reaction and enhances the performance of the developed imprinted polymers [16,17].

Based on the idea of using DMSO and RTIL as a binary solvent and β-CD as a functional monomer, MIPs for the selective extraction of FNT were developed, and the polymerization ratio was optimized. The prepared polymers’ efficiency was assisted with different parameters, such as kinetic adsorption and an adsorption isotherm, and structural and morphological characterization was also carried out. The MIPs were applied to assist in fenthion detection in real samples, including soil, lettuce and grapes, using HPLC-UV.

## 2. Materials and Methods

### 2.1. Chemicals and Reagents

FNT (>95%), abamectin (ABM) (97%), 1-butyl-3-methylimidazolium tetrafluoroborate ([BMIM]BF_4_) (>98%) and 1,6-hexamethylene diisocyanate (HMDI) (>98%) were supplied by TCI (Shanghai, China). DMSO (Superdry) (≥99.9%), methyl parathion (MP) (98%), acetamiprid (ACP) (97%) and β-CD (>98%) were obtained from J&K Tech. Ltd. (Beijing, China). Before utilization, the β-CD was dried at 110 °C. The soil samples were collected from the local experimental field of Guangxi University (Nanning, China). The lettuce and grape samples were purchased from the local market of Nanning (Guangxi, China) and stored at −20 °C. The Macklin Biochemical Technology Co., Ltd. (Shanghai, China) provided acetic acid, methanol, acetone and acetonitrile (all HPLC grade). All the reagents used were of analytical grade.

### 2.2. Instruments Used

The different instruments used in this research were as follows: Zeiss SUPRA 55-VP with a field emission electron gun (FEG), scanning electron microscope (SEM) (Oberkochen, Germany), TGA/DSC 1/1600 (Mettler Toledo, Schwerzenbach, Switzerland), Milli-Q IQ 7000 (Merck, Darmstadt, Germany), Tristar II 3020 (Micromeritics, Norcross, GA, USA), DF-101D magnetic stirrer (Yuhua, Changzhou, China), BY-400C centrifuge (Bai-yang Medical Inst., Beijing, China), Heraeus Vacutherm VT 6060M (Thermo Electron, Langenselbold, Germany), 0.22 µm, 13 mm nylon filter (Bojin, Tianjin, China), HZQ-F160A thermostatic oscillator (Shanghai Yiheng scientific instruments, China), ultrasonic cleaner GT SONIC-D20 (Guangdong, China) and vortex mixture Mix-30S (MIU Instruments, Hangzhou, China).

### 2.3. Chromatographic Analysis

The HPLC Agilent^®^ Model 1260 Infinity II provided by Agilent Technologies (Santa Clara, CA, USA) was used for chromatographic analysis, which was equipped with an ultraviolet (UV) detector and binary pump (600 bar). The analysis was carried out using the method established by Wu et al. [4], with some modifications. The mobile phase comprised methanol: deionized water (8:2 *v*/*v*) (degassed for 30 min before use), with a flow rate of 1 mL min^−1^, column temperature of 30 °C and injection volume of 20 µL. The FNT was detected at a wavelength of 220 nm, using the ZORBAX Eclipse Plus column (C18, 95 Å, 4.6 × 250 mm, 5 µm) for separation. The analysis was carried out on the Agilent OpenLAB Chromatography Data System software (Santa Clara, CA, USA) and OriginPro, Version 2021 (OriginLab Corporation, Northampton, MA, USA).

### 2.4. Preparation of Molecularly Imprinted Polymers

The imprinted polymers were synthesized according to Zhao et al. [16], with some modifications. The functional monomer (β-CD) and template (FNT) were stirred in a binary solution of RTIL ([BMIM]BF_4_) and DMSO in a 3 n flask until completely dissolved under constant N_2_ purging. The mixture was shifted to a silicon oil bath, and the temperature was adjusted to 65 °C. Afterwards, the cross-linker (HMDI) was added quickly, and the polymerization was carried out for 10 h. The crude polymers were precipitated in acetone overnight. The extraction of the template was carried out with methanol: acetic acid (8:2 *v*/*v*) using an ultrasonic method, and the obtained samples were dried at 50 °C. The non-imprinted polymers (NIPs) used as controls were synthesized via a similar protocol, but the addition of the template was excluded. Table 1 shows the production process for MIPs synthesized at different ratios.

### 2.5. MIP Characterization Analysis

To check the porosity of the prepared imprinted polymers, a swelling experiment was carried out according to Ying et al. [18]. Briefly, 100 mg of each polymer was packed in a graduated syringe (1 mL), which was filled with deionized water and was left for 12 h at 25 °C. After the required time, excess water was removed, and the MIPs were analyzed according to the following equation.

$$Sr=\frac{We-Wi}{Wi}$$

where *Sr* represents the total swelling ratio, and the weights of the swollen polymer and dry polymer are expressed as *We* and *Wi*, respectively.

The SEM, operated at an accelerated voltage of 2 kV, was used to analyze the polymers’ structure, and thermogravimetric analysis (TGA) at room temperature to 800 °C was performed to determine the thermal stability of the prepared polymers. The carrier gas was nitrogen and the temperature rate was 10 °C per minute.

### 2.6. Adsorption Isotherm

Different concentration ranges from 10 to 100 mg L^−1^ in methanol were used to assist the FNT binding with the prepared polymers. In detail, 50 mg of each polymer (M1, N1, M10, N10, individually) was added to each standard solution of FNT (5 mL) and agitated for 30 min at 25 °C. After this, the sample was centrifuged at 7000 rpm for 3 min to separate the polymer layer from the solution. For the evaluation of the required equilibrium concentration (*Q_e_*), expressed as (mg L^−1^), the supernatant was filtered with a 0.22 nylon filter and was subjected to HPLC–UV to analyze the remaining concentration. The *Q_e_* was calculated according to Farooq et al. [11] using the following equation.

$$Q_{e}=\frac{\left(C_{0}-C_{e}\right)\times V}{m}$$

where “*V*” (mL) represents the volume of each sample solution used; “*m*” denotes the mass of the polymer used for each sample; and the initial and equilibrium concentration of FNT are expressed as “*C_0_*” and “*C_e_*”, respectively.

### 2.7. Adsorption Kinetics

Different time intervals in the range of 5–120 min were used to assist the FNT binding with the prepared polymers. In detail, 50 mg of each polymer (M1, N1, M10, N10, individually) was added to a standard solution of FNT (5mL sample volume, 50 mg L^−^^1^ FNT concentration) and agitated for different time intervals at 25 °C. After the specified time, the sample was centrifuged at 7000 rpm for 3 min to separate the polymer layer from the solution. For the evaluation of the required adsorption amount at the specified time (*Q_t_*), expressed as (mg g^−1^), the supernatant was filtered with a 0.22 nylon filter and was subjected to HPLC–UV to analyze the remaining concentration. The *Q_t_* was calculated according to the following equation.

$$Q_{t}=\frac{\left(C_{0}-C_{i}\right)\times V}{m}$$

where “*V*” (mL) represents the volume of each sample solution used; “*m*” denotes the mass of the polymer used for each sample; and the initial and final concentration of FNT are expressed as “*C_0_*” and “*C_i_*”, respectively.

### 2.8. Selective Recognition Experiment

To investigate the specific recognition of MIPs towards FNT, three competitor compounds, MP, ACP and ABM, were selected for the selectivity test. First, 50 mg of MIP or control polymer (M1, N1, M10, N10, individually) was added to a 50 mg L^−1^ solution of each analyte separately. All the samples were agitated at an equilibrium time at 25 °C. Then, the polymers were removed from the sample by centrifugation, and the supernatants were filtered before HPLC–UV analysis to determine the remaining concentrations of the solutions.

### 2.9. Extraction of Template

The efficiency of MIPs is greatly affected by template removal because incomplete removal will lead to lower adsorption of the analytes. In this experiment, the ultrasonication method was used for template extraction from the polymer matrix by using different types of eluents. Three types of eluents, methanol: acetic acid, acetone: acetic acid and hexane: acetic acid at a ratio of 9:1 and 8:2 *v*/*v* were used. The prepared polymers were added to 10 mL of each eluent and were sonicated for 5 min. After each cycle, a rotatory evaporator was used to concentrate the supernatant, and it was reconstituted in methanol. After filtration with a 0.22 µm filter, HPLC–UV was used to determine the extracted template amount until no template residue was found in the supernatant. To remove the residual acetic acid, methanol or acetone was used, and the obtained polymers were vacuum-dried at 50 °C.

### 2.10. Reusability

The continuous adsorption experiments were performed with 5mL of 50 mg L^−1^ methanol standard solution of FNT and 50 mg of the MIP (M1 and M10, individually) was added to each cycle. The samples were agitated for 30 min for the adsorption of FNT by the polymer. The polymer was separated from the supernatant by centrifugation and then 10 mL of methanol: acetic acid (8:2 *v*/*v*) was added to the polymer and sonicated for 5 min. The rotatory evaporator was used to concentrate the supernatant and it was reconstituted in methanol. After filtration with a 0.22 µm filter, HPLC–UV was used to determine the extracted FNT amount.

### 2.11. FNT Detection in Real Samples

Three types of real samples, soil, lettuce and grapes, were selected for this study to determine the MIPs’ suitability for FNT detection in real samples. The lettuce and grape samples were thoroughly washed and homogenized with a blender. All the samples were pre-analyzed before MIP addition to confirm the absence of FNT. Then, 20 g of each sample was spiked with an FNT standard solution of 0.05, 0.1 or 0.20 mg L^−1^. To separate the phases, 4 g of MgSO_4_ and 3 g of NaCl were added, shaken and centrifuged at 9000 rpm for 3 min. Then, 50 mg of MIP (M1) was added to the upper methanol layer after transferring it to another vial and the sample was agitated for 30 min to achieve the equilibrium adsorption of FNT by MIPs. The polymer was separated from the sample through centrifugation, and the adsorbed FNT was eluted from the MIP with methanol: acetic acid (8:2 *v*/*v* 10 mL). The supernatant was concentrated, reconstituted in methanol, filtered with a 0.22 µm filter and analyzed with HPLC–UV.

## 3. Results and Discussion

### 3.1. Polymer Synthesis

In this research, we prepared MIPs based on β-CD for the detection of FNT by using different ratios of [BMIM]BF_4_ (RTIL) as a cosolvent with DMSO (Table 1). The adsorption results reveal that the MIPs prepared with an RTIL: DMSO ratio of 1:2 provide higher adsorption of FNT while increasing the [BMIM]BF_4_ amount, resulting in the decreased adsorption of MIPs. The polymerization ratio 1:4:16 (template, functional monomer and cross-linker, respectively) showed the highest adsorption of FNT (Figure S1). The functional monomer and cross-linker ratio of 1:4 provided the highest adsorption capacity for fenthion due to the linkage between the OH group of cyclodextrin and an NCO group of an isocyanate, while the ratios of 1:2 and 2:5 provided lower adsorption capacity [19]. Among evaluated polymerization timings (4–24 h), 10 h is sufficient for MIP synthesis. Using DMSO as a solitary solvent does not provide sufficient support for polymerization [20]. Using RTIL as a cosolvent significantly enhances and supports the polymerization reaction and promotes favorable interactions of fenthion and β-CD due to its tunable nature [16,21]. In contrast, a higher amount of RTIL significantly reduces the MIPs’ efficiency, which might be attributed to the imidazolium cation (BMIM+) interaction with β-CD through the butyl group [22]. Based on the results, M1 and M10 with representative control polymers (N1 and N10) were selected for further experiments.

### 3.2. Characterization Study

The results showed that M1 had the lowest swelling performance, followed by M10, while the highest swelling was observed in both control polymers (Figure S2). Thus, using RTIL as a cosolvent for MIPs leads to a more porous structure because of the low swelling due to the water molecules embedded in the cavity. The lower swelling of polymers clearly supports MIPs’ adsorption ability [23]. Ying et al. [18] also corroborate the results by demonstrating that high MIP efficiency is allosteric to lower swelling.

**Figure 2:**
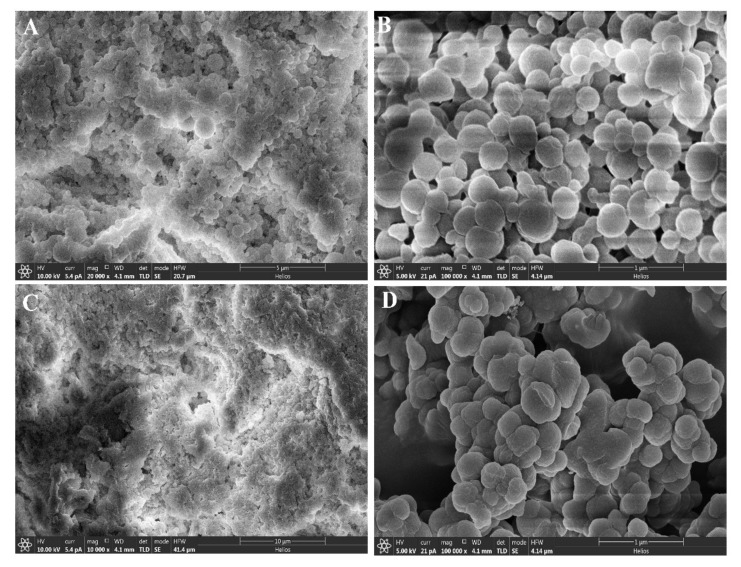
SEM images of (**A**) N1, (**B**) M1, (**C**) N10, (**D**) M10.

From the results of SEM analysis, it can be seen that M1 had a highly porous structure compared to the corresponding non-imprinted polymers and M10, and the average diameter of the particles was less than 500 nm (Figure 2). In comparison, M10 also had a less porous structure compared to M1 as it was a neat DMSO-based polymer. Both control non-imprinted polymers had a compact structure when compared to the corresponding imprinted polymers. The more porous structure of MIPs contributed to efficient MIP synthesis as the porosity was induced with template removal [24].

**Figure 3:**
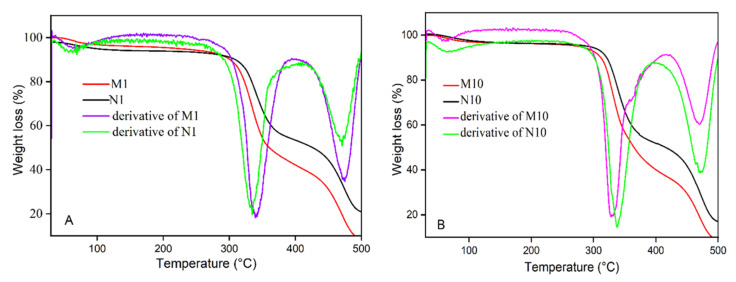
(**A**) TGA curves of M1 and N1, (**B**) TGA curves of M10 and N10.

TGA analysis was performed to evaluate the thermal stability of the imprinted polymers under a nitrogen atmosphere. As shown in Figure 3, the results of the TGA curves demonstrated that the MIPs possessed high thermal stability and the polymers were stable up to 300 °C, and after that the decomposition had occurred. Both imprinted polymers (M1 and M10) followed a similar trend, and the first section of the curve represented water loss or evaporation of the solvents from the polymers [25]. The second section of the curve at around 300 °C indicated that the composite decomposition and the complete decomposition occurred at 400 °C [26].

### 3.3. Adsorption Isotherm

**Figure 4:**
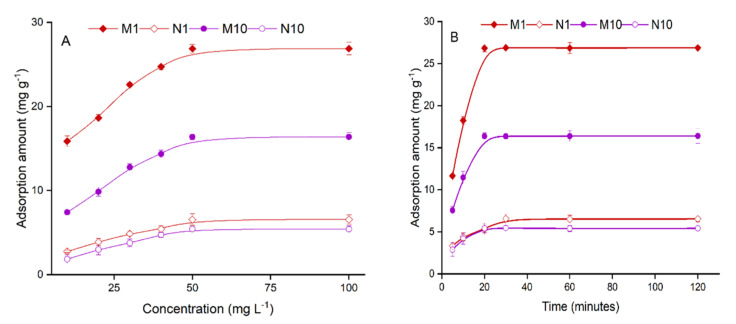
(**A**) Isothermal binding of the polymers (adsorption time: 30 min), (**B**) Kinetic adsorption of the polymers (concentration: 50 mg L^−1^, temperature 25 °C).

The results of isotherm binding are given in Figure 4A, which shows that the adsorption of FNT by the polymers was gradually increased with an increase in the concentration of FNT and reached equilibrium at a 50 mg L^−1^ concentration of FNT. To assess the results of isotherm binding of the polymers, the Langmuir and Freundlich isotherm models [24] were applied according to the following equations.

$$\frac{C_{e}}{Q_{e}}=\frac{1}{K_{L}Q_{max}}+\frac{C_{e}}{Q_{max}}$$

$$lnQ_{e}=lnK_{f}+\frac{1}{n}lnC_{e}$$

where the equilibrium adsorption amount and equilibrium adsorption concentration of FNT are expressed as *Q_e_* (mg g^−1^) and Ce (mg L^−1^), respectively. The maximum adsorption capacity of the polymers is indicated as “*Q_max_*” (mg g^−1^) and can be derived from a linear plot of *C_e_/Q_e_* versus *C_e_*. *K_L_* represents the Langmuir isotherm constant, and *K_f_* represents the Freundlich isotherm constant, both related to the affinity of the binding sites and expressed as (L mg^−1^). The values of the Freundlich isotherm model, namely “*K_f_*” and “*n*”, can be derived from the plot of *lnC_e_* versus *lnQ_e_*. The results revealed that the highest theoretical adsorption amount, “*Q_max_*” (29.70 mg g^−1^), was obtained by M1, while the M10 had a “*Q_max_*” of 19.35 mg g^−1^. Compared with the Freundlich isotherm model, the Langmuir isotherm model yielded the highest correlation coefficient (R^2^) values for all polymers and therefore fitted well (Table 2), indicating that the MIPs achieved monolayer chemical adsorption [27]. Based on the highest “*Q_max_*” value, R^2^ value (0.997) and *K_L_* value of 0.010 (L mL^−1^), M1 had better results than other polymers, while the polymers prepared with only DMSO as a solvent (M10) showed poorer results as compared to M1. The linear fitting plot results of both the Langmuir and Freundlich isotherm models are given in Figures S3 and S4.

### 3.4. Adsorption Kinetics

The prepared polymers showed a rapid adsorption response to FNT as rapid adsorption was achieved in the first 10 min, reaching an equilibrium position in a total of 20 min. M1 had the highest adsorption capacity of 26.85 mg g^−1^ at 20 min, followed by M10 with an adsorption amount of 16.37 mg g^−1^ (Figure 4B). However, the N1 polymers showed slower adsorption of 6.56 mg g^−1^ in an equilibrium time of 30 min. The “pseudo-second-order kinetic model” was used to determine the kinetic adsorption of the prepared polymers, as given in the following equation.

$$\frac{t}{Q_{t}}=\frac{1}{kQ_{e}^{2}}+\frac{t}{Q_{e}}$$

where *Q_e_* represents the total adsorption amount at the equilibrium time, and *Q_t_* represents the FNT adsorbed (mg g^−1^) at the specific time “*t*”. “*k*” represents the pseudo-second-order rate constant, expressed as (mg g^−1^ s^−1^). The R^2^ values for each polymer can be determined from the linear fitting plot of *t/Q_t_* versus *t* (Figure S5). The M1 polymers exhibited a *Q_e_* value of 28.01 (mg g^−1^), which was higher than that of neat DMSO-based polymers (M10), and had an R^2^ value of 0.998 (Table 2). The adsorption process of all the polymers was governed by chemisorption [28], as indicated by the highest correlation coefficient values of each polymer, which indicate that the pseudo-second-order kinetic model fitted well in the study.

### 3.5. Selectivity

**Figure 5:**
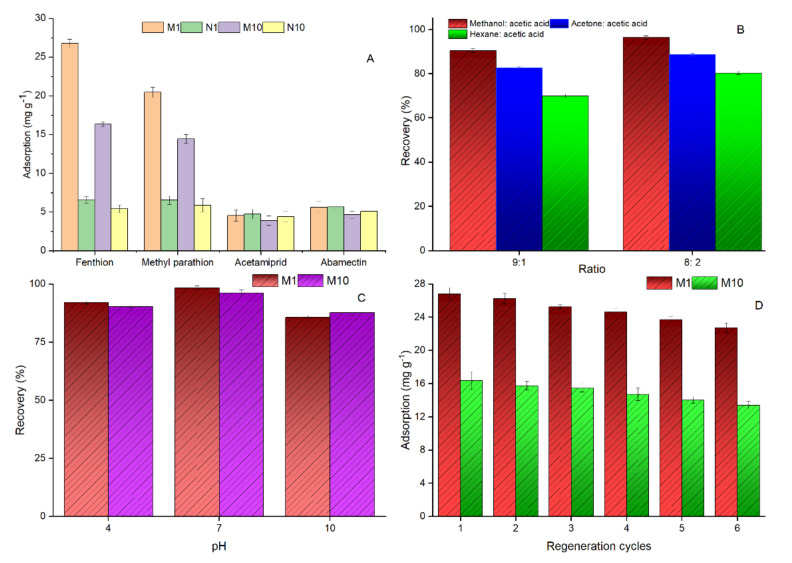
(**A**) Selectivity parameters of MIPs for fenthion and its competitors, (**B**) Removal of template recovery (%) with different eluents, (**C**) Adsorption response of MIPs at different pH levels, (**D**) Adsorption recoveries of fenthion by MIPs in regenerated cycles.

For the experiment on the recognition ability of MIPs towards FNT, three competitor compounds, methyl parathion, acetamiprid and abamectin, were selected (Figure 1). The results showed that M1 achieved higher adsorption for FNT (26.80 mg g^−1^), but it showed cross-selectivity towards methyl parathion, with an adsorption capacity of 20.44 mg g^−1^ (Figure 5A). M10 followed a similar trend to M1, but its adsorption ability was lower in comparison with M1. The selectivity of the polymers was further assessed with the imprinting factor (*IF*), which is expressed as below.

$$IF=Q_{MIP}/Q_{NIP}$$

where *Q_MIP_* and *Q_NIP_* refer to the adsorption capacity of MIPs and NIPs, respectively, expressed as (mg g^−1^). The *IF* values for FNT and methyl parathion were M1: 4.07, 3.12 and M10: 3.02, 2.46, respectively, while the *IF* values for acetamiprid and abamectin were M1: 0.96, 0.99 and M10: 0.88, 0.92, indicating that M1 has higher adsorption efficiency for FNT.

### 3.6. Template Extraction

For the removal of FNT molecules from the polymer matrix, the ultrasonication method was used because it requires a short time, and less eluent is used when compared to traditional Soxhlet extraction [29]. The results showed that methanol: acetic acid provided better template extraction than acetone: acetic acid, and 96.40% template recovery was achieved using methanol with 20% acetic acid, while, at the same ratio, 88.71% recovery was achieved when methanol was replaced with acetone (Figure 5B). The use of hexane: acetic acid provided lower FNT extraction from the crude polymer matrix. The amount of acetic acid significantly altered the template elution. From the results, it can be seen that 10% acetic acid provided the lowest elution of FNT. The acetic acid weakened the bond between template and polymer that provides rapid template extraction; besides this, it also minimized the amount of eluent [30].

### 3.7. pH Optimization

In analytical methods, the extraction of analytes from complex samples is significantly affected by the pH of the sample medium. FNT is usually stable at neutral pH, while it degrades at alkaline pH. Based on the nature of FNT, the adsorption efficiency of MIPs towards FNT was investigated in three types (4, 7 and 10) of pH buffer solution. The results reveal that the lowest recovery of 85.74% (M1) and 87.67% (M10) was achieved at an alkaline pH level of 10 (Figure 5C). The highest recovery of 98.41% (M1) and 96.23% (M10) was obtained at pH 7, followed by pH 4, with a total recovery of 92.17% (M1) and 90.36% (M10). These results are in agreement with those of Gao et al. [2], who found that the recovery of FNT is lower at an alkaline pH.

### 3.8. Reusability

The applicability, reliability and cost-effectiveness of the designed MIPs are notably dependent on the reusability of the polymers [31]. To improve the potential re-application ability of the synthesized MIPs, a reusability experiment was carried out for both M1 and M10 (Figure 5D). In a total of six continuous regeneration cycles, 15.27% and 18.02% loss were observed in M1 and M10, respectively. Both polymers M1 and M10 showed less than 6% loss in the first three cycles, reaching approximately 10% in 4 continuous regeneration cycles. Overall, M1 showed a 3% lower performance loss than M10 in all six regeneration cycles. The nature of the inclusion interaction between guest and host molecules affects the regeneration performance of MIPs.

### 3.9. FNT Detection in Real Samples

For the purpose of exploring the reliability and applicability of the prepared polymers (M1) for the detection of FNT, three different types of samples, soil, lettuce and grapes, were selected. FNT standard solutions in the linear range of 0.02–3.00 mg g^−1^ were used for method validation before the analysis. From the linear fitting plot of the concentration versus calibration curve of peak area, an R^2^ value of 0.999 was achieved. As seen in Table 3, the results showed that a good recovery was achieved in all three samples. The recovery of FNT in soil was lower (87.44–93.00%) when spiked with three different levels of FNT. The highest recovery of 101.25% and 99.11% was attained at a spiked level of 0.20 mg L^−1^ in grapes and lettuce, respectively. The limit of detection (LOD) and limit of quantitation (LOQ) were 0.04 mg L^−1^ and 0.11 mg L^−1^, respectively, with a relative standard deviation (RSDs) lower than 3%. The results demonstrate that the application of synthesized MIPs provides excellent potential for the detection of FNT in real samples conforming with analytical standards. A comparative study was carried out between the developed method and the previously published method for the detection of FNT (Table 4). The comparative study shows that the presented work provides a useful analytical tool for FNT detection in real sample analysis.

## 4. Conclusions

This research work was based on the synthesis of MIPs using β-CD as a functional monomer for FNT detection based on applying RTIL as a cosolvent to improve the affinity of the polymers. For the synthesis of MIPs, the polymerization ratio and the amounts of RTIL and DMSO were optimized. The successful synthesis of polymers was evaluated through morphological and structural investigations. The results showed that the polymers formed using RTIL as a cosolvent have a good adsorption ability of 26.85 mg g^−1^, with an adsorption equilibrium time of only 20 min; however, the polymers showed cross-selectivity to methyl parathion. From the analysis of soil, lettuce and grapes, the polymers had good recovery rates of 87.44–01.25%, with RSDs lower than 3% and an LOD of 0.04 mg L^−1^. Based on these results, including their good adsorption ability, reusability, easy preparation and high stability, this research work proposes a new standard analytical approach for the rapid detection of FNT in real samples.

## Figures and Tables

**Table 1:** Protocols of MIPs synthesis for fenthion.

Polymers	Fenthion (mmol)	β-CD (mmol)	HMDI (mmol)	RTIL (mL)	DMSO (mL)
M1 *	1	4	16	10	20
M2	1	4	16	15	15
M3	1	4	16	20	10
M4	1	8	20	10	20
M5	1	8	20	15	15
M6	1	8	20	20	10
M7	1	12	24	10	20
M6	1	12	24	15	15
M9	1	12	24	20	10
M10 *	1	4	16	0	20

* NIPs were prepared for the mentioned polymers.

**Table 2:** Langmuir, Freundlich isotherms models and Pseudo-second-order parameters for the binding of FNT on MIPs and NIPs.

Langmuir Isotherm Model			
Polymers	*Q*_max_ (mg g^−1^)	*K_L_* (L mL^−1^)	R^2^
M1	29.70	0.010	0.997
N1	7.92	0.287	0.992
M10	19.35	0.041	0.994
N10	6.96	0.485	0.984
**Freundlich isotherm model**			
Polymers	*N*	*K*_f_ (L mg^−1^)	R^2^
M1	3.905	9.089	0.946
N1	2.461	1.164	0.955
M10	2.650	3.322	0.949
N10	1.201	0.660	0.944
**Pseudo-second-order model**			
Polymers	Q_e_ (mg g^−1^)	*K* (mg g^−1^ s^−1^)	R^2^
M1	28.01	0.010	0.998
N1	6.83	0.034	0.999
M10	17.01	0.018	0.999
N10	5.56	0.075	0.999

**Table 3:** Determination of fenthion in real samples.

Sample	Spiked Levels (mg L^−1^)	Found Concentration (mg L^−1^)	RSD (%)	Recovery (%)
Soil	0.20	0.186	1.61	93.00
	0.10	0.091	2.08	91.20
	0.05	0.044	2.80	87.44
Lettuce	0.20	0.198	1.31	99.11
	0.10	0.097	1.95	97.41
	0.05	0.047	2.11	94.64
Grapes	0.20	0.203	1.28	101.25
	0.10	0.099	2.23	98.77
	0.05	0.048	2.30	95.60

**Table 4:** Comparison of the present work with previously reported methods for FNT detection.

Sample	Method	Recovery (%)	RSD (%)	LOD (mg L^−1^)	LOQ (mg L^−1^)	Linear Range (mg L^−1^)	Reference
Olive oil	MIP-HPLC-UV	96.1	–	0.005	0.023	–	[3]
Water and apple	MIP-HPLC-UV	94.0–100.4	1.7–4.2	0.0018	–	0.02–10	[2]
Lettuce	MSPE-HPLC-UV	89.2–101.2	9.1	0.0005	0.0015	0.0015–2.0	[4]
Lettuce	SPE HPLC-PDA	96.0–104.2	6.2	0.006	0.02	0.02–0.40	[32]
Fruits	GC-FPD	91–112	3.7	0.000033	0.00198	0.0001–0.1	[33]
Urine	SPE HPLC-UV	92.69–95.64	3.75	0.00458	–	0.02–0.12	[34]
Soil, lettuce and grapes	MIP-HPLC-UV	87.44–101.25	1.28–2.80	0.04	0.11	0.02–3.0	This work

## Data Availability

Not applicable.

## Supplementary Materials

The following supporting information can be downloaded at: https://www.mdpi.com/article/10.3390/nano12132129/s1, Figure S1: Adsorption data of the synthesized polymers for fenthion; Figure S2: Swelling performance of the polymers; Figure S3: Linear fitted plots of Ce/qe versus Ce for Langmuir isotherm model; Figure S4: Linear fitted plots of log q versus log Ce for Freundlich isotherm model; Figure S5: Linear fitting of the plot of t/Q versus t.
