# Supplementary material for: Development of Molecularly Imprinted Polymers for Fenthion Detection in Food and Soil Samples
Source: Nanomaterials (Basel). 2022 Jun 21;12(13):2129. doi: 10.3390/nano12132129 (PMC9268004; doi:10.3390/nano12132129)
Supplement: Supplementary file 1 [file nanomaterials-12-02129-s001.zip › nanomaterials-1748418-supplementary.pdf]

nanomaterials

MDPI

Article

Development of Molecularly Imprinted Polymers for Fenthion Detection in Food and Soil Samples

Saqib Farooq, Bochang Chen, Fukun Gao, Ihsan Muhammad, Shakeel Ahmad, and Haiyan Wu*

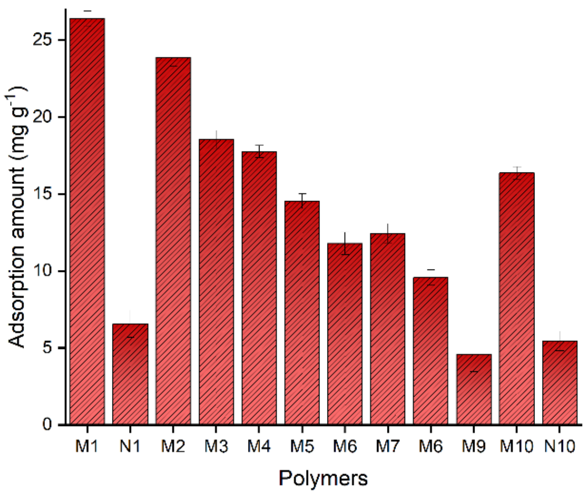

Figure S1. Adsorption data of the synthesized polymers for fenthion.

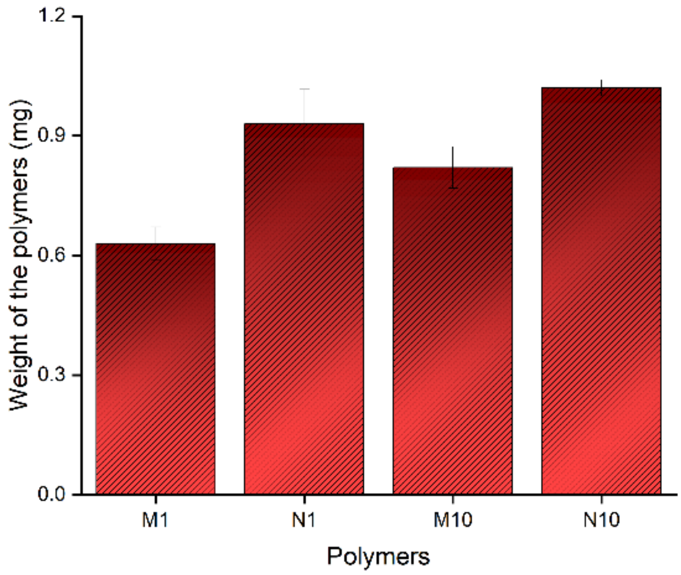

Figure S2. Swelling performance of the polymers.

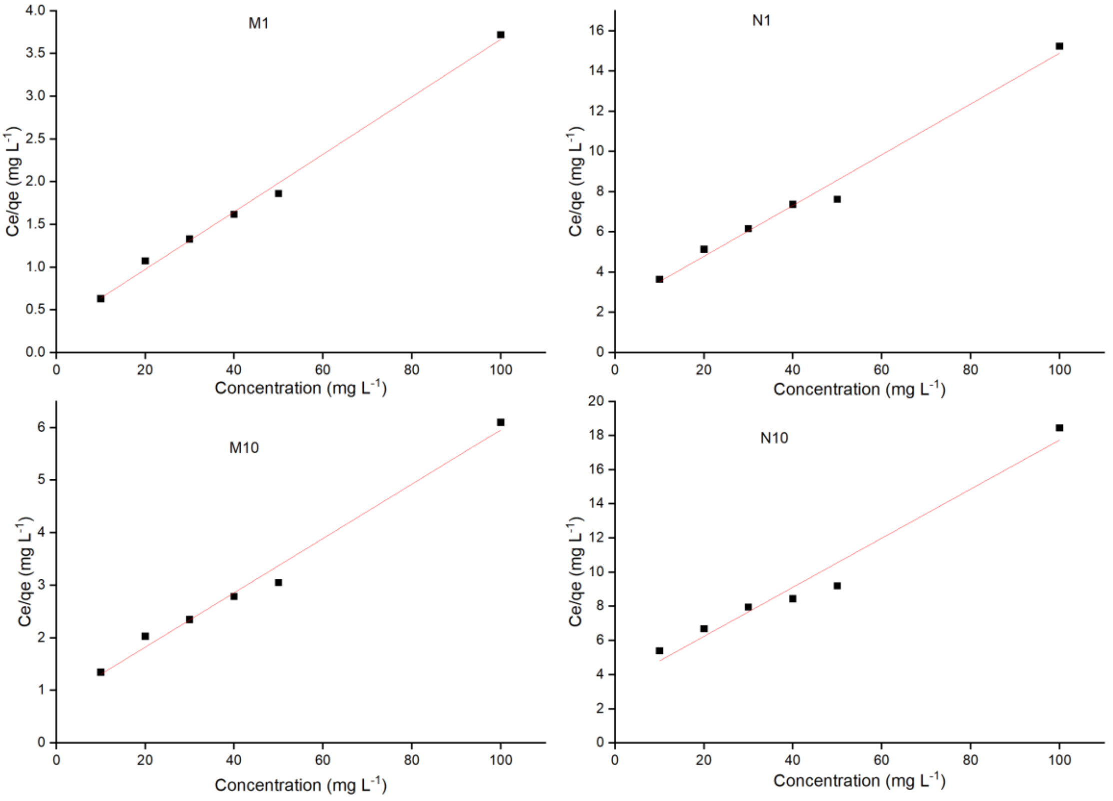

Figure S3. Linear fitted plots of Ce/qe versus Ce for Langmuir Isotherm model.

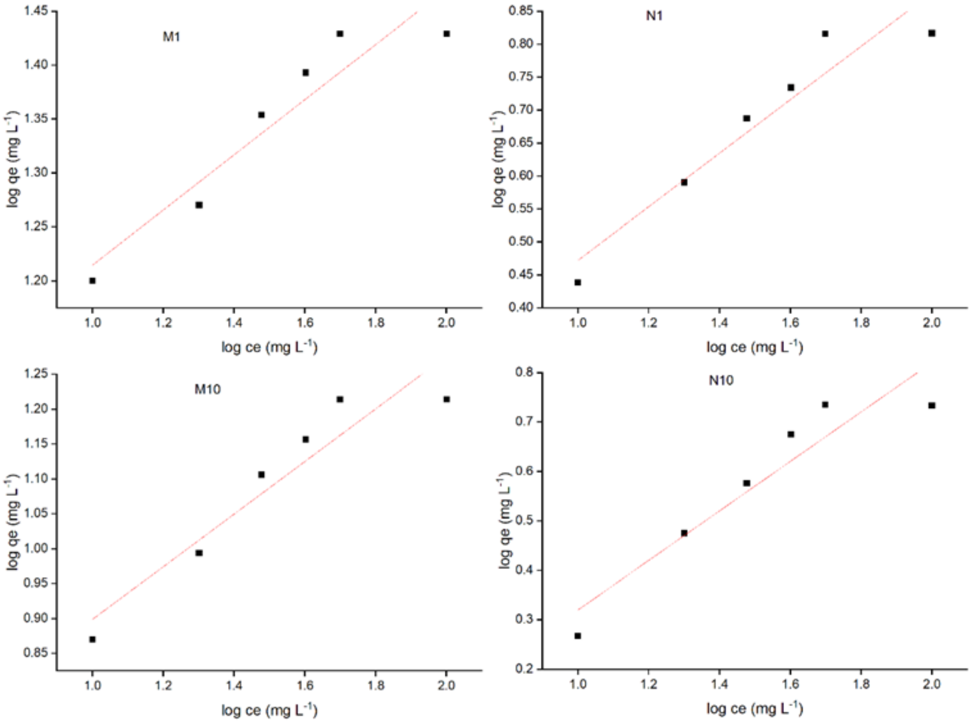

Figure S4. Linear fitted plots of log q versus log Ce for Freundlich Isotherm model.

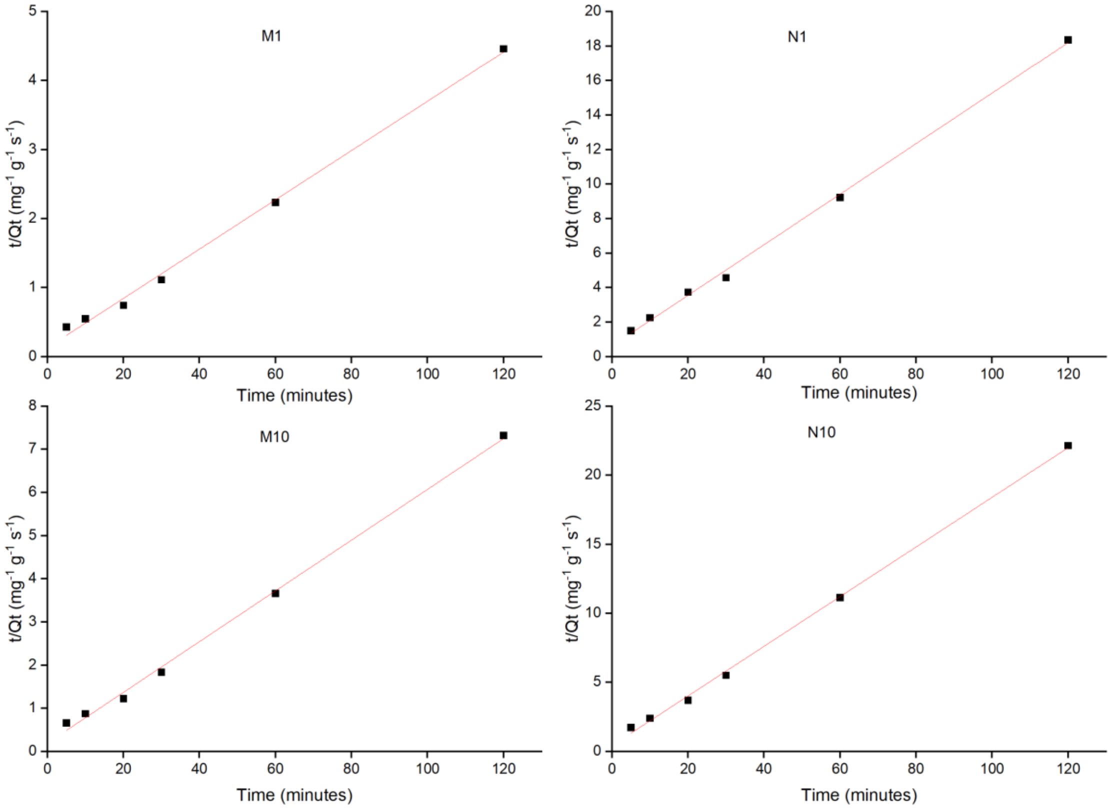

Figure S5. Linear fitting of the plot of t/Q versus. t.
